# Small and simple: next-generation miniaturized diffraction-based spectrometer with computational reconstruction algorithms

**DOI:** 10.1038/s41377-024-01449-7

**Published:** 2024-05-07

**Authors:** Markus Suta

**Affiliations:** Inorganic Photoactive Materials, Faculty of Mathematics and Natural Sciences, Heinrich Heine University Düsseldorf, Düsseldorf, Germany

**Keywords:** Imaging and sensing, Optoelectronic devices and components

## Abstract

An ultra-simple and miniaturized spectrometer using an arbitrarily shaped pinhole as diffraction element reconstructs a broadband spectrum from the information of diffraction of monochromatic radiation by clever computational reconstruction algorithms. This circumvents complex calibration procedures and paves the way to cost-effective on-chip spectrometers combining fast acquisition without significant loss in spectral resolution.

Spectrometers are easily set-up and offer an ever-modern way to investigate the interaction between light and matter. In principle, they consist of a light source, a dispersing element, and a light-sensitive detector. In most modern simple optical spectrometers, the dispersing elements for white-light sources are blazed or holographic gratings. As diffraction requires apertures with lateral spacings in the order of the wavelength of the electromagnetic radiation of interest and spectral resolution is governed within the far-field (Fraunhofer) limit, there is a general limitation for miniaturization referred to as the footprint of a spectrometer. Moreover, the diffraction efficiency and light throughput of grating optics is not constant over the whole spectral range and especially drops quickly below a blazing wavelength. For fast spectral acquisition, the diffracted light is directed on two-dimensional (2D) array detectors such as complementary metal oxide semiconductor (CMOS) or charged-couple device (CCD) cameras that assign each wavelength to a pixel and thus, can acquire a spectrum faster than conventional photon counting systems such as a photomultiplier tube. Alternative approaches that circumvent diffractive optics make use of 2D bandpass filter arrays right on top of the detector arrays such as quantum dot absorbers based on their size-dependent wavelength-dispersive absorption properties^1–3^.

An approach that has gained a lot of attention due to constant improvements in digital signal processing and, more recently, even machine learning is reconstruction-based spectrometry^4–8^. It relies on the fact that the diffracted pattern of light from an object projected on a 2D detector array does formally allow to reconstruct the original intensity pattern from the diffraction pattern. A similar—although not precisely the same—problem arises in structure determination by single-crystal X-ray diffraction originally exploited over a century ago by William Henry Bragg and his son, William Lawrence Bragg^9^. For that type of diffraction, the periodic array of point scatterers (i.e., the crystal structure) is reconstructed from the 2D projected diffraction pattern based on an accurate measurement of the intensities and angles of the different diffraction spots. For that purpose, monochromatic X-ray radiation (usually Cu *K*_*α*_ or Mo *K*_*α*_) is used. By the same computational advancements, such a structural refinement can now be even elaborated on crystalline powders, i.e., randomly oriented sets of microcrystallites that do not give rise to diffraction spots but whole circles on a 2D array. Reconstruction of the structure is possible for an educated guess of the structural motif within the crystallites and a least-squares procedure minimizing the difference between expected intensities of the diffraction circles to the actually measured ones. This problem has been originally tackled by Hugo Rietveld and has now become a standard to refine structures from powdered, crystalline compounds based on the diffraction of monochromatic X-ray light^10^.

The diffraction pattern does not only contain information about the structural details of the object that diffracts but also about the originally used point light source. Thus, if a particularly simple diffraction object is used, similar least-squares methodologies should allow to reconstruct the spectral profile of a light source from the diffraction pattern. While the angular intensity distribution of diffracted light over the pixels of a 2D array detector is measurable and easily connected to the wavelength *λ* of the incident light in the Fraunhofer diffraction limit knowing the distance *z* between diffraction spot and detector (in the Fraunhofer limit, it is *z* ≫ *λ*), the power spectrum, *ω*(*λ*), of the used light source is much more challenging to reconstruct. In a recent publication in *Light: Science & Applications*, Chuangchuang Chen, Honggang Gu, and Shiyuan Liu have faced this problem and found an elegant solution that now paves the way to miniaturized, yet diffraction-based spectrometers using a reconstruction algorithm^11^.

The authors used a small pinhole with a diameter in the order of the wavelength of incident light. For an idealized circular aperture, it is known from optics that the intensity distribution of a monochromatic point light source, *I*_m_(*θ*), with wavelength *λ* due to diffraction at the circular aperture of radius *r* in the far-field Fraunhofer limit at the diffraction angle *θ* is given by the Airy pattern^12^,

1 $${I}_{{\rm{m}}}\left(\theta ,\lambda \right)={I}_{0}{\left(\frac{{J}_{1}({kr}\sin \theta )}{{kr}\sin \theta }\right)}^{2}$$

where *J*_1_(*x*) is the Bessel function of the first kind and *k* = 2π/*λ* is the wavenumber. The brightest spot is in the middle (*θ* = 0), while it is followed by concentric disks due to mutual interference of the evolving spherical wavefronts at the circular aperture. For longer wavelengths, the spreading *θ* between the diffraction maxima is larger, which is a simple way to disperse white light at a circular aperture given that the detector is located at sufficiently long distances compared to the wavelength of the incident light.

While a circular aperture would offer a very simple way to set up a miniaturized spectrometer, a key obstacle is, in fact, that the intensity distribution for the employed white-light source (represented by *I*_0_ in Eq. (1)) is not constant but also wavelength-dependent, i.e., *I*_0_ = *I*_0_(*λ*). Moreover, the diffraction efficiency of the pinhole may not be the same for every wavelength. Finally, the pinhole may not be perfectly circular, but could be more arbitrarily shaped, which leads to more complicated diffracted intensity profiles than shown in Eq. (1). Due to the latter case, the diffracted intensity profile of a point light source is generally referred to as a so-called point spread function, PSF(*θ*, *λ*). Usually, this is not known a priori, but needs to be approximated very well, or directly measured by diffraction of a well-defined monochromatic coherent point light source. The diffracted intensity profile of a broadband light source, *I*_b_(*θ*), is then the incoherent sum of the various point spread functions for each chosen wavelength *λ*_1_, …, *λ*_*n*_ acting on the original, discretized power spectrum ω(*λ*) of the light source,

2 $${I}_{{\rm{b}}}\left(\theta \right)=\int {\rm{d}}\lambda \,\omega \left(\lambda \right){\rm{PSF}}\left(\theta ,\lambda \right)\approx \mathop{\sum }\limits_{j=1}^{n}\left({\lambda }_{j}-{\lambda }_{j-1}\right)\omega \left({\lambda }_{j}\right){\rm{PSF}}\left(\theta ,{\lambda }_{j}\right)$$

since the integration time of the detector is usually longer than the coherence time of the broad-band source. Upon discretization of Eq. (2) and restriction to selected wavelengths, a linear system of equations results that can be recast in matrix form,

3 $${\boldsymbol{b}}={\bf{A}}{\mathbf{\omega}}$$

with ***b*** as the *n* × 1 column vector containing the broadband diffraction intensity distributions at chosen wavelengths *λ*_1_, …, *λ*_*n*_, **A** as the (*M* ∙ *N*) × *n* matrix representing the point-spread functions weighted with the wavelength increments projected on the *M* × *N* pixel array of the detector, and ***ω*** as the *n* × 1 column vector containing the power spectrum for each of the chosen wavelengths. Formally, the spectrum can be recovered if Eq. (3) is inverted or deconvolved. A analogous example from e.g., photography or microscopy is filtering of images to enhance their resolution. By that virtue, Eq. (2) is central to the action of an arbitrarily shaped pinhole to make it work for a miniaturized spectrometer. The clever trick that Chen, Gu, and Liu have now relied on in their publication in *Light: Science & Applications* is that the point spread function can be transferred to all desired wavelengths in the visible range given that it is has been measured for one selected wavelength *λ*_m_ of a monochromatic, coherent light source, i.e., PSF(*θ*, *λ*_*j*_) ≈ PSF(*θ*, *λ*_m_)^11^. Under this assumption, the demands on an explicit shape of the pinhole can be completely released. Their conceptual approach is summarized in Fig. 1.

**Fig. 1:**
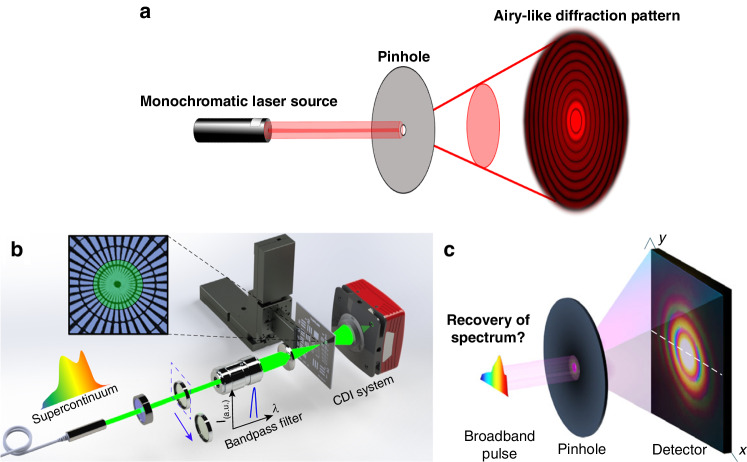
Diffraction-based miniaturized spectrometer employing computational reconstruction. **a** Diffraction of monochromatic light at a circular aperture. In the Fraunhofer (far-field) limit, this gives rise to the well-known Airy disks. **b** Concept of presented one-to-broadband diffraction-based reconstruction spectrometry at a pinhole demonstrated by Chen et al.^11^. If the diffraction patterns from selected monochromatic wavelengths are used as input, the original broadband spectrum can be recovered by reconstruction algorithms from the diffraction pattern at an arbitrarily shaped pinhole. **c** Ultimate schematic idea of the proposed computational reconstruction technique proposed by Chen et al.^11^

Several practical limitations pose problems to straightforward usage of Eq. (3). One issue in the measurement of a point spread function is that it necessarily contains noise. Especially for large diffraction angles *θ* this can be a problem, for which the intensities of the diffraction profile and thus, the point spread function are usually low. Inversion of Eq. (3) is then said to be ill-defined and some entries of ***ω*** can become highly sensitive to slightest changes in the low-intensity diffraction data. One can cope with this mathematical issue by so-called regularization and definition of constraints, which is mandatory to still allow for reasonable reconstruction of the power spectrum encoded in the vector ***ω***. The authors went for an appealingly simple approach of a Tikhonov–Phillips regularization well-known from machine-learning approaches^13,14^. In this technique, noisy or small entries of matrix **A** in Eq. (3) (and thus, the point spread function) are enhanced by a regularization matrix **Γ**, which has to be chosen wisely to balance robustness of the deconvolution of Eq. (3) and resolution. By additionally convolving the thus obtained raw power spectral data with a so-called Hann window that particularly suppresses noise and data scatter at the boundaries, the authors could refine the data quality even more.

Next to this fundamental problem, another concern is the finite detector array size of the employed CMOS or CCD camera that determines the overall achievable spectral resolution. This is usually referred to as footprint-resolution dilemma. An ingenious and yet simple way the authors went for to fully exploit the limited sensor array size and thus, its dynamic range with simultaneous use of as many diffraction orders as possible was overexposure of the detector and usage of a suited filter for the central brightest spot (cf. Eq. (1)) to enhance the signal-to-noise ratio of the higher diffraction orders. They then compared the increase in computational costs upon increase of the sensor area and number of exploited pixels to the gain in spectral resolution and figured out that 1024 pixels offer a good compromise leading to a spectral resolution of 1 nm. Several other corrections such as for the quantum efficiency of the detector and non-linearities of the detection unit additionally help increase the accuracy of the reconstruction procedure. Finally, the authors nicely showed how important the quality of the input data is—a key to all machine-based retrieval and learning algorithms. Since the decomposition in Eq. (2) relies on the input of point spread functions from a monochromatic light source used to reconstruct the spectrum of a broadband source, a crucial factor for the overall spectral resolution in the reconstructed spectrum is the actual monochromaticity of the employed training light source, i.e., the spectral width of the quasi-monochromatic light source used in diffraction.

By only a limited number of simple and intuitive approximations, the authors have demonstrated how to overcome the footprint-resolution dilemma and combined modern computer-assisted reconstruction techniques with the yet most established way of dispersing white light with high spectral resolution: diffraction. It will be interesting to see how the evolution of spectroscopy will continue and what applications can emerge out of that. Besides the simple miniaturization of the spectrometer footprint for on-chip photonic technologies, also coherent diffractive X-ray imaging has become a possible application to overcome the limitation of required crystalline, 3D periodic structures and offer structural insights despite the lack of any long-range order^15,16^. It could also change the field of recently Nobel Prize-awarded attosecond spectroscopy^5,17–22^, for which temporal resolution comes at the price of required spectrally broad pulses. Thus, any simultaneously high spatial resolution for, e.g., analysis of electron dynamics at the nanoscale requires a miniaturized broadband imaging technique, which could have now come a bit closer by the authors’ work in *Light: Science & Applications*.
